# Butterfly gene flow goes berserk

**DOI:** 10.1186/s13059-016-0898-z

**Published:** 2016-02-27

**Authors:** Richard H. Ffrench-Constant

**Affiliations:** Biosciences, University of Exeter, Tremough, Penryn TR10 9EZ UK

## Abstract

A new study shows that genomic introgression between two *Heliconius* butterfly species is not solely confined to color pattern loci.

Please see related Research article: www.dx.doi.org/10.1186/s13059-016-0889-0

## Introduction

A group of animals that cannot interbreed with animals from another group is often taken as the definition of a ‘true’ species. However, a suite of recent studies suggests that the rare event of hybridization, that is, the production of viable offspring by the interbreeding of individuals from two such species, can facilitate adaptation through the process of genome ‘introgression’. Thus, a region of the genome that encodes a potentially advantageous phenotype, such as a novel color pattern, can be acquired wholesale, and transferred from one species to the other.

Adaptation through genome introgression is well documented in plants [1], but examples from animals are scarce. Two examples from animals come from the sharing of pesticide-resistance genes. The first is in Old World mice who share genes that confer resistance to the anticoagulant rodenticide warfarin [2]. The second is the exchange of a mutation that is associated with insecticide resistance between closely related species of the malarial mosquito *Anopheles*, following the deployment of insecticide-treated bed nets for mosquito control [3]. It is therefore tempting to speculate that such dramatic acquisitions of large portions of the genome can only be driven by traits that are under strong natural selection. In the examples above, it is thus easy to imagine the substantial selective advantage conferred by the recently transferred resistance genes upon exposure to a pesticide or insecticide. Nevertheless, other, more classic examples of natural selection, such as variation in beak shape in Darwin’s finches (which is driven by a single transcription factor ALX1 that affects craniofacial development), also seem to be driven by genomic introgression [4] and here the selective forces are presumably more modest.

In this context, *Heliconius* butterflies are a unique model system in which to examine the relative importance of genomic introgression. This is because they have color patterns that are involved in Mullerian mimicry and hence dramatically increase their relative fitness against predation. These color patterns seem to have resulted from both introgression between closely related species and convergent evolution between more distantly related species. Thus, *Heliconius* species within the *melpomene-cydno-timareta* clade have been shown to share wing color patterns that have resulted from genomic introgression [5], whereas other more distantly related species pairs, such as *H. melpomene* and *H. erato*, appear to have evolved similar wing patterns independently. The relative importance of genomic introgression in swapping color patterns among other *Heliconius* species remains unclear. New findings presented by Wei Zhang and colleagues [6] in *Genome Biology* suggest, however, that signatures of genome introgression in *Heliconius* species extend far beyond color patterns alone, and call into question what biological functions other introgressed loci are performing.

## Introgression in the postman

To examine the relative importance of introgression, Wei Zhang and colleagues analyzed a silvaniform species, *H. besckei*, that still shares the striking red and yellow bands of the ‘postman’ color pattern with *H. melpomene nanna*, with which it flies in coastal Brazil. They developed a comprehensive statistical pipeline for looking at genome-wide patterns of divergence and introgression between the different *Heliconius* species using full genome re-sequencing data [6]. This pipeline integrates both population genetic and phylogenetic approaches to infer introgression, and has significant power because candidate regions are examined using more than a single statistic. To define a sister taxon for *H. besckei*, they first examined the evolutionary history of *Heliconius* using 23 million single nucleotide polymorphisms (SNPs). This analysis allowed the authors to select *H. besckei*, *H. numata* and *H. m. nanna* as the three ingroup taxa for Patterson’s *D*-statistic tests, which are used to detect introgression. They then examined the simple hypothesis that color patterns had been shared between species either from *H. melpomene* to *H. besckei* or the other way around.

The red and yellow bands of the postman pattern are controlled by the loci *B*/*D* and *Yb*, respectively, allowing Wei Zhang and colleagues to focus on two candidate regions of the genome already shown to be involved in introgression. Interestingly, they found strong evidence for introgression of the red *B*/*D* locus between *H. besckei* and *H. m. nana* but little support for sharing of the yellow *Yb* locus between the two. This may suggest that the *Yb*-encoded yellow pattern may have evolved independently in these two species. At the *B*/*D* locus, the region showing a strong introgression signal was upstream of the gene *optix*, a transcription factor that controls the morphology and color of the scales in the red bands [7]. This region was not within the *optix* open reading frame (ORF) itself, a finding that highlights the resolving power of the authors' statistical test for identifying potential regulatory elements controlling key color-pattern genes. A total of 85 candidate introgression loci passed a sequencing coverage test that looked for evidence of false positives in the datasets. Of these, 41 were supported by all the different statistical measures when overlaid onto the genome.

## Beyond color

Of the 41 candidate loci, 32 appeared to contain protein-coding genes. This supports the hypothesis that protein-coding genes, as well as regulatory elements, have also been swapped to confer novel functions. The authors list a range of potential functions for these ORFs, including the production of collagen, cuticle matrix formation, metabolism, embryonic patterning, synaptic function and heat stress. It is difficult to see from candidate gene functions alone, however, how the transfer of such regions can confer any adaptive advantage to the recipient. One potential exception in this long list of candidate genes is a genomic region on chromosome 1 that appears to have been transferred alongside the *B*/*D* mimicry locus from *H. melpomene* to *H. besckei* and is flanked by genes encoding type IV collagen subunits α-1 and α-2. These two type IV collagen subunits have recently been linked to enhanced flight muscle function in migratory forms of the monarch butterfly, *Danaus plexippus* [8]. As mimicry in *Heliconius* is thought to involve both changes in color pattern and flight behavior [9], Wei Zhang and colleagues suggest the fascinating hypothesis that introgression of the locus neighboring these two collagen subunit genes may also change the flight behavior of the recipient butterfly, so that it more closely mimics the flight behavior of other distasteful butterflies, thus further reducing predation. Thus, changes to both butterfly color and flight behavior may have introgressed together to improve mimicry in the recipient.

## Improving dating and sex

In addition to providing more information on loci that are likely to have undergone introgression in butterflies, Wei Zhang and colleagues also used their improved statistical pipeline to look at introgression on the butterfly sex chromosome. Butterflies and moths are female heterogametic (ZW), and the Z chromosome is thought to play an important role in hybrid sterility in *Heliconius* [10]. Comparison of *H. m. nanna* with other silvaniform species, such as *H. ethilla*, showed an unexpected pattern of introgression on the Z chromosome. Further, this pattern could be traced back to the predicted exchange of the entire Z chromosome between one silvanifrom subclade and the ancestor of the entire *melpomene-cydno-timareta* clade. As Haldane’s rule predicts that hybrid sterility should affect the heterogametic sex and because recessive Z-linked loci have indeed been shown to cause hybrid sterility in *Heliconius* [10], this suggests that genes controlling hybrid sterility may have been introgressed from one group of butterflies to another while contained within the Z chromosome.

Finally, Wei Zhang and colleagues are keen to emphasize that their improved analysis of introgression allows us to look at the timing and fate of introgressed loci. They argue that a locus that increases fitness in butterflies, such as a mimicry locus, should be expected to spread and potentially become fixed in that species. Neutral or deleterious alleles at this locus should then undergo random recombination and accumulate further substitutions, decreasing the probability that they will become fixed. Perhaps more importantly, the physical length of the introgressed haplotypes can also tell us how recent the gene flow was and can provide information on the strength of selection on the introgressed DNA. In short, the authors argue that the small, introgressed genomic regions resulting from the oldest events are likely to represent the most adaptive and highly selected events.

## Challenges for the future

This improved statistical pipeline for genome-wide screening for introgression is likely to impact other fields beyond the study of butterflies. For example, how many other classic textbook examples of natural selection are also the result of introgression? What about melanism in butterflies and moths? Is this also the result of exchanges of entire loci between species and is this the same in both butterflies and moths? What percentage of pesticide-resistance mutations have arisen de novo and how many have simply been swapped between closely related species? Like any good study, this work clearly raises more questions for future work. What seems certain, however, given the widespread availability of SNP data for many species, is that we should see the role of adaptive introgression examined across many more classic examples of natural selection.

## Abbreviations

ORF: Open reading frame
SNP: Single nucleotide polymorphism
